# Development of transgenic rats producing human β-amyloid precursor protein as a model for Alzheimer's disease: Transgene and endogenous APP genes are regulated tissue-specifically

**DOI:** 10.1186/1471-2202-9-28

**Published:** 2008-02-26

**Authors:** Cansu Agca, Jason J Fritz, Lary C Walker, Allan I Levey, Anthony WS Chan, James J Lah, Yuksel Agca

**Affiliations:** 1 University of Missouri College of Veterinary Medicine, Department of Veterinary Pathobiology Columbia, MO 65211, USA; 2 Department of Neurology and Center for Neurodegenerative Disease, Emory University, Atlanta, GA 30322, USA; 3 Yerkes National Primate Research Center, Emory University, Atlanta, GA 30329, USA

## Abstract

**Background:**

Alzheimer's disease (AD) is a devastating neurodegenerative disorder that affects a large and growing number of elderly individuals. In addition to idiopathic disease, AD is also associated with autosomal dominant inheritance, which causes a familial form of AD (FAD). Some instances of FAD have been linked to mutations in the β-amyloid protein precursor (APP). Although there are numerous mouse AD models available, few rat AD models, which have several advantages over mice, have been generated.

**Results:**

Fischer 344 rats expressing human APP driven by the ubiquitin-C promoter were generated via lentiviral vector infection of Fischer 344 zygotes. We generated two separate APP-transgenic rat lines, APP21 and APP31. Serum levels of human amyloid-beta (Aβ)_40 _were 298 pg/ml for hemizygous and 486 pg/ml for homozygous APP21 animals. Serum Aβ_42 _levels in APP21 homozygous rats were 135 pg/ml. Immunohistochemistry in brain showed that the human APP transgene was expressed in neurons, but not in glial cells. These findings were consistent with independent examination of enhanced green fluorescent protein (eGFP) in the brains of eGFP-transgenic rats. APP21 and APP31 rats expressed 7.5- and 3-times more APP mRNA, respectively, than did wild-type rats. Northern blots showed that the human APP transgene, driven by the ubiquitin-C promoter, is expressed significantly more in brain, kidney and lung compared to heart and liver. A similar expression pattern was also seen for the endogenous rat APP. The unexpected similarity in the tissue-specific expression patterns of endogenous rat APP and transgenic human APP mRNAs suggests regulatory elements within the cDNA sequence of APP.

**Conclusion:**

This manuscript describes the generation of APP-transgenic inbred Fischer 344 rats. These are the first human AD model rat lines generated by lentiviral infection. The APP21 rat line expresses high levels of human APP and could be a useful model for AD. Tissue-specific expression in the two transgenic rat lines and in wild-type rats contradicts our current understanding of APP gene regulation. Determination of the elements that are responsible for tissue-specific expression of APP may enable new treatment options for AD.

## Background

Alzheimer's disease (AD) is a devastating neurodegenerative disorder that affects 7–10% of elderly individuals over 65 years of age and nearly 50% of those over 85 in the U.S [1]. AD also can be inherited in an autosomal dominant manner, which causes a familial form of AD (FAD) that usually emerges at younger ages than does idiopathic AD [2]. FAD has been linked to mutations in the β-amyloid precursor protein (APP) [3], as well as presenilin 1 [4] and presenilin 2 [5], which are critical components of the γ-secretase complex that liberates the amyloid-β peptide (Aβ) from membranes. Aβ is the major proteinaceous component of senile plaques, and all known FAD mutations increase the production of Aβ or its tendency to aggregate. Two predominant forms of Aβ, Aβ_40 _and Aβ_42_, result from the proteolytic cleavage of APP by β- and γ-secretases [6]. Because it is highly amyloidogenic, Aβ_42 _is believed to play a particularly important role in the pathogenesis of AD [7].

Several transgenic (Tg) mouse models of AD have been generated to study the effects of APP mutations. The genetic background of mice has been shown to have a significant effect on plasma and brain Aβ_40 _and Aβ_42 _levels as well as on Aβ-deposition in brain [8]. Outbred mouse lines show variability in Aβ production and deposition, whereas Aβ production is more consistent within an inbred transgenic line. In this regard, the generation of transgenic rats on various genetic backgrounds (*i.e. *inbred Lewis and Fischer 344 strains) has been more difficult than mice and outbred rats (*i.e. *Sprague Dawley and Wistar) [9]. Recently, outbred single-transgenic rats have been generated that express either human wild type [10] or mutant APP [11,12]. Transgenic Wistar rats overexpressing human APP695 (hAPP695) were used to investigate the role of APP in the recovery process following cerebral ischemia [10]. The authors report that despite the total APP/Aβ levels in cortex and hippocampus of hAPP695 rats being twice the level of non-transgenic controls, hAPP695 did not develop amyloid plaques with aging. Tg Wistar rats overexpressing the APP_Sw/Ind _transgene showed some intraneuronal Aβ immunoreactivity but failed to develop β-amyloid deposits by 24 months of age [11]. Recently, Folkesson et al. [12] reported the development of an outbred SD rat line expressing APP_Sw _which begin to show extracellular Aβ that is predominantly found in cerebrovascular blood vessels beginning at 15 months of age. Ruiz-Opazo et al. [13] reported generation of inbred APP_Sw _Fischer-344 rats with a 56.8% increase in APP mRNA expression in the brain of transgenic animals compared to non-transgenic cohorts at 12-months of age. The APP_Sw _Fischer-344 rats did not show evidence of extracellular amyloid deposits or senile plaques up to 18 months of age. Interestingly, APP_Sw _Fischer-344 rats showed attenuated hippocampus-dependent learning and memory decline compared to non-transgenic cohorts as measured by Morris Water Maze. Ruiz-Opazo [13]and colleagues have postulated that within a particular expression range APP or its derivatives my play a role in normal learning and memory, but when expressed at levels exceeding this threshold APP and/or its metabolites could lead to neuronal loss and cognitive decline.

Although mice have been extensively used in many areas of biomedical research, rat models have certain advantages over mice due to their larger size, unique genetics, and well-studied behavioral characteristics [14]. Rats are better suited for microsurgery, cell and tissue transplantation, *in vivo *functional analyses, and studies that require multiple sampling [15]. In the context of neurological studies, stereotaxic injection is a commonly used method that is easier to perform with precision in rats than in mice. For these reasons, it is advantageous to have germline Tg rat models of neurodegenerative diseases like AD in order to better understand the underlying biochemical changes taking place during disease development and to test potential treatment options. The AD rat models developed previously were generated mostly on outbred rat strains. Our aim was to develop an inbred APP transgenic rat line with high APP expression in the brain, as we believe this may be one of the important prerequisites for producing amyloid pathology in rat models of AD.

## Results

### Promoter Characterization and Selection

**Figure 1:**
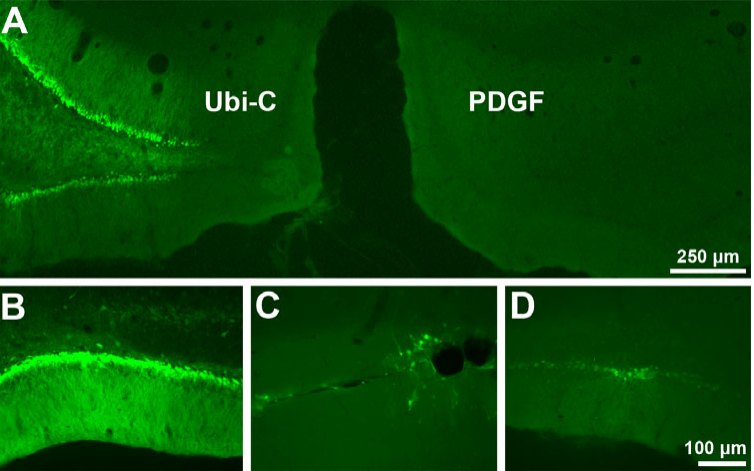
Comparison of promoters following lentivirus injection in rat brain. Lentiviruses were stereotaxically injected into rat hippocampus and examined after three months. **A**. eGFP expression driven by the ubiquitin-C promoter (Ubi-C) was consistently superior to that of other promoters, including the platelet-derived growth factor promoter (PDGF) and cytomegalovirus. Lower panels show representative higher power images of eGFP driven by Ubi-C- (**B**), cytomegalovirus- (**C**), and PDGF- (**D**) promoters in lentivirus/eGFP-injected rats.

Comparison of enhanced green fluorescent protein (eGFP) expression driven from ubiquitin-C (Ubi-C), cytomegalovirus (CMV), or platelet-derived growth factor (PDGF) promoters in SD rat brains after stereotaxic injection of lentivirus showed that the Ubi-C promoter yielded consistently superior eGFP expression than did the CMV-eGFP and PDGF-eGFP viruses. Figure 1 shows eGFP expression in rat brains three weeks post-injection of Ubi-C-eGFP and CMV-eGFP lentiviruses. Enhanced GFP expression driven by CMV and PDGF promoters appeared to decrease dramatically over time. In contrast, strong Ubi-C- eGFP expression persisted without apparent diminution at the longest time-point examined (13 months, data not shown). These results with Ubi-C are consistent with previous findings using the Ubi-C promoter in driving transgene expression [16].

**Figure 2:**
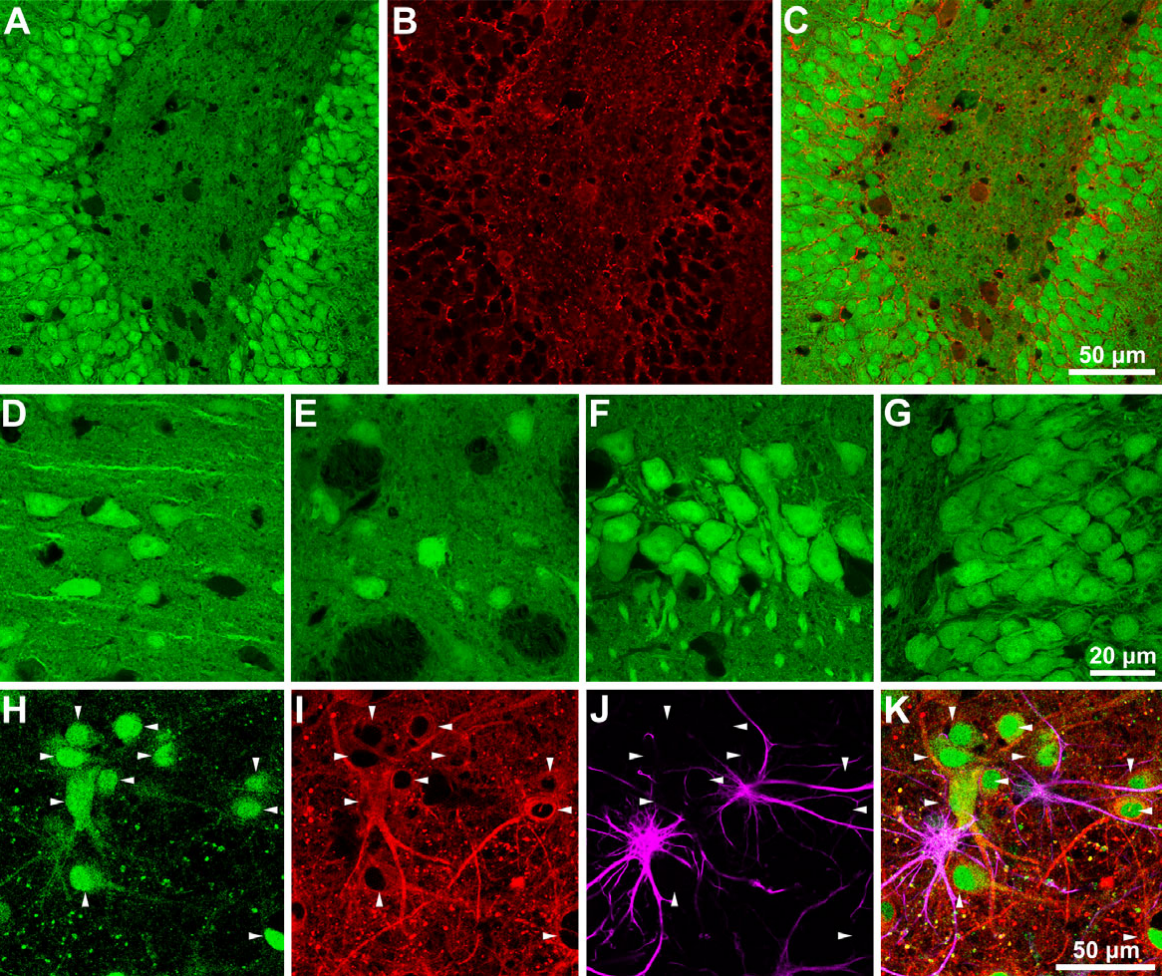
eGFP expression in the brain of a transgenic rat (Ubi-C promoter). Upper panel: eGFP expression is restricted to neurons in transgenic rat brain. **A**. Confocal microscopy shows extensive eGFP expression in dentate granule cells. **B**. GFAP immunoreactivity reveals astroglial cells. **C**. Merged images show a lack of colocalization of eGFP and GFAP signals. Middle panel: confocal images reveal robust eGFP expression in neuronal cell bodies. **D**. Cortex. **E**. Striatum. **F**. CA1 region of hippocampus. **G**. Hippocampal dentate gyrus. Lower panel: Mixed primary cultures from E18 embryos were stained with anti-beta III tubulin to identify neurons (**I**; red) and GFAP to label glial cells (**J**; purple). eGFP expression in cultured neurons was concentrated in the nucleus as well as the cytoplasm (**H**; green). Arrowheads denote individual neurons in all panels, and the merged image (**K**) shows eGFP in neurons but not in glial cells.

While stable, high-level transgene expression is highly desirable in Tg animals, neuron-selective expression is also an important consideration in selecting an optimal promoter. Brain expression of the eGFP transgene driven by a Ubi-C promoter was examined in Tg SD rats created by injection of eGFP-lentivirus into fertilized zygotes. Strong eGFP expression was seen in adult rats; by confocal microscopy, eGFP expression appeared to be restricted to neurons (Fig. 2). When eGFP distribution was directly compared to immunostaining with glial fibrillary acidic protein (GFAP) to label astrocytes, there was strong divergence in the staining patterns. To confirm the apparent restriction of eGFP driven by the Ubi-C promoter to neurons, primary cultures were established from E18 Tg SD embryos and employed for colocalization studies using markers for neurons and glial cells. Confocal images of mixed primary cultures stained with neuron-specific beta tubulin and GFAP is shown in Figure 2J. Enhanced GFP is expressed in neurons, but not in glial cells, confirming the neuronal specificity of eGFP expression driven by the Ubi-C promoter.

### Transgenic Founders

Table 1 summarizes the Tg rates following lentiviral delivery of the APP_Sw/Ind _double mutant construct. We injected a total of 168 zygotes, transferred 156 of them to recipient foster mothers, and obtained 18 live pups as a result. Both PCR and Southern analysis revealed 4 Tg founder pups carrying the APP_Sw/Ind _double mutant transgene. The Tg rate based on the numbers of Tg pups born/numbers of embryos transferred was 22% (4/18). Aβ ELISA assessment revealed that two (APP21 and APP23) of these 4 founders had detectable levels of human Aβ_40 _in serum samples by 9 weeks of age (140 and 126 pg/ml, respectively).

**Table 1:** Transgenic efficiency after injection of lentiviral vectors carrying the APP_Sw/Ind _double mutation under control of the ubiquitin-C promoter in Fischer 344 rat zygotes.

Zygotes injected	Embryos transferred	Live pups born	Transgenic pups	Germline transgenic
168	156 (93%)	18 (12%)	4 (22%)	2 (50%)

### Initial Screening of APP-Transgenic Founders and Generation of Homozygous Transgenic Rats

**Figure 3:**
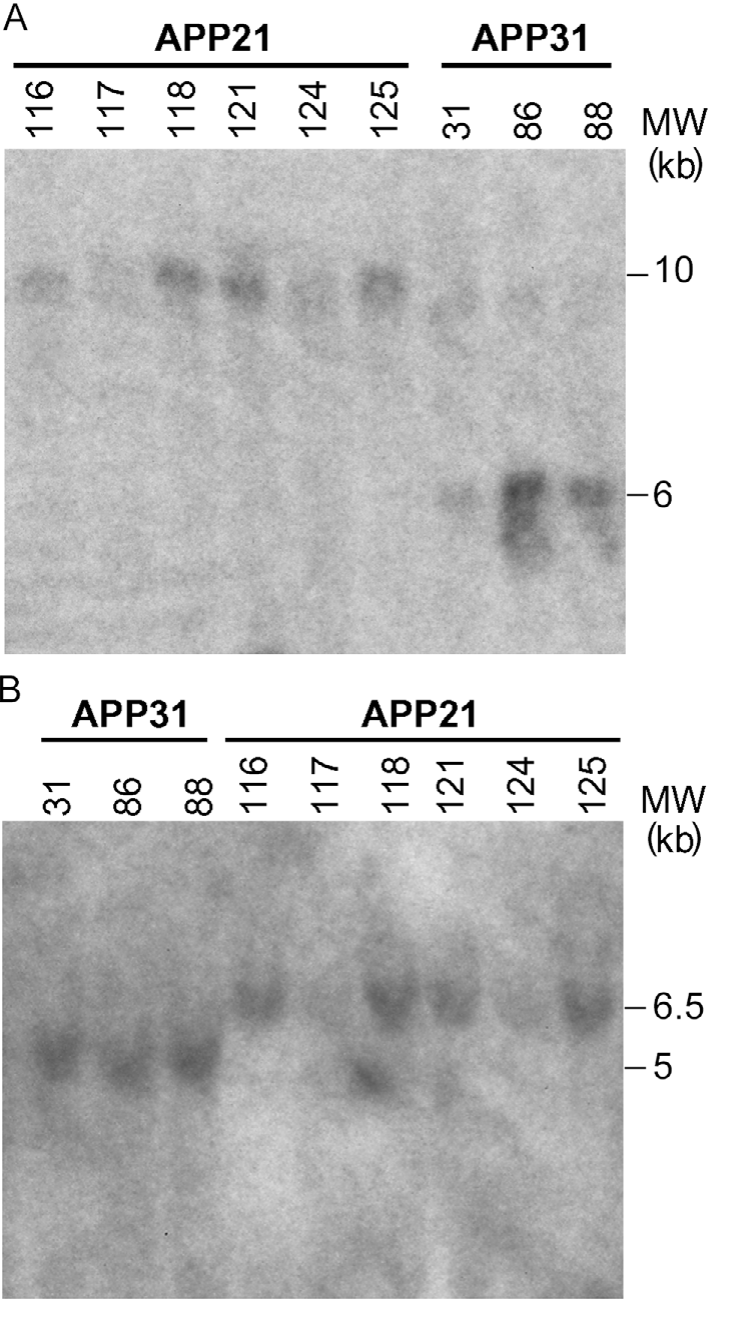
Southern blot hybridization of genomic DNA obtained from APP21 and APP31 rats. **A**. Genomic DNA from APP21 (116, 117, 118, 121, 124, 125) and APP31 (31, 86, 88) animals were digested with BamHI and hybridized with a human APP probe. **B**. Genomic DNA from APP31 (31, 86, 88) and APP21 (116, 117, 118, 121, 124, 125) were digested with EcoRI and hybridized with a human APP probe.

Four PCR- and Southern blot-positive APP_Sw/Ind _Tg rats were generated (Fig. 3). Two of the founders were germline Tg (APP21 and APP31) and each had a single copy of the transgene. The other two were not germline Tg (APP23 and APP30) and each contained two copies of the transgene. The molecular weights of DNA fragments containing the transgene that were obtained through BamHI digestion were 10 and 6 kb for APP21 and APP31 lines, respectively. EcoRI digestion of genomic DNA yielded 6.5 and 5 kb fragments for APP21 and APP31 lines, respectively. Both APP21 and APP31 Tg lines contained one copy of the transgene as determined by two separate restriction enzyme digestions (EcoRI and BamHI; Fig. 3). Different molecular weights of the transgenes in APP21 and APP31 animals is due to different transgene integration sites in these two transgenic lines. Both APP21 and APP31 lines were bred for five generations, demonstrating the stability of the transgene across generations; both lines appear normal and do not have any indication of unpredicted side effects of the transgenes.

### APP Transgene Expression

**Figure 4:**
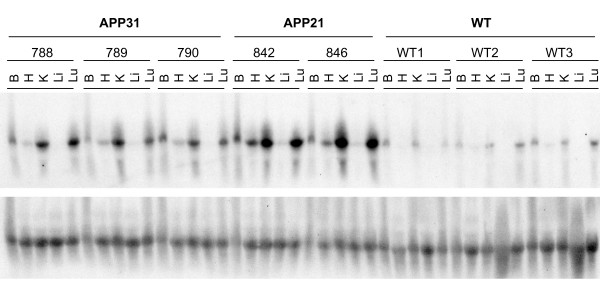
Northern blot hybridization of total RNA from tissues of the APP31 and APP21 lines as well as WT rats. Total RNA hybridized with the human APP probe (upper autoradiogram) prior to hybridization with 18S rRNA (lower autoradiogram). B: Brain, H: Heart, K: Kidney, Li: Liver, Lu: Lung.

**Figure 5:**
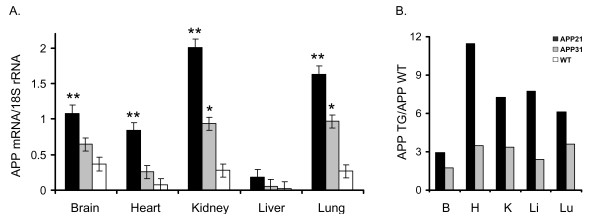
Gene expression differences among organs obtained from APP21, APP31 and WT rats. **A**. The expression of APP genes is normalized by dividing the net intensity of APP bands by the 18S rRNA bands. ** represent significantly greater APP expression in APP21 compared to APP31 and WT rats (P < 0.05); * represents significantly greater APP expression in APP31 compared to WT rats (P < 0.05). B. APP expression-difference between APP21 and APP31 rats compared to WT animals. The values were obtained by dividing the APP expression in each organ (B: Brain, H: Heart, K: Kidney, Li: Liver, Lu: Lung) by APP expression in WT rats.

Expression of the APP transgene was determined by Northern blot analysis in homozygous rats (Fig. 4). The APP probe used for Northern blot analysis hybridizes both the human APP transgene as well as native rat APP. The BLAST score (NCBI, Bethesda, MD) of the 773 bp human APP probe template to rat APP mRNA was 547. The molecular weight of the APP mRNA was approximately 2.5 kb. Northern blot analysis showed that the APP21 line expressed the highest levels of APP mRNA, the APP31 line expressed intermediate levels (Fig. 4 and 5A), and the native rat APP mRNA expression was the lowest. Expression of APP showed a tissue-dependent variation. The transgene was highly expressed in kidney and lung of both the APP21 and APP31 lines. Expression in brain was intermediate, and expression in liver and heart was the lowest for both Tg lines (Fig. 4 and 5A). Similar expression patterns for rat APP mRNA were observed in non-transgenic rats, such that liver and heart contained lower APP compared to brain, kidney and lung. The average expression among the organs analyzed was 4.3 times lower in WT rats than in APP Tg rats. Brain APP expression in the APP21 line was 1.7 and 2.9 times greater than in APP31 and WT rats, respectively (Fig. 5B).

### Aβ ELISA

Serum levels of human Aβ_40 _were determined in homozygous APP21 (n = 2), APP31 (n = 3), and hemizygous APP21 (n = 16) rats. In addition, 26 rats from two Tg parents, denoted as 'homozygosity status unknown', were included in the statistical analysis (Table 2). Serum Aβ_40 _was measurable in all APP21 animals, and homozygous rats had 1.6 times greater Aβ_40 _than did hemizygous rats. Average serum Aβ_40 _levels of the APP31 line were 5 times lower than those of the APP21 line. In addition, Aβ_40 _was undetectable in 10 out of 20 rats, which includes 3 homozygous rats. Serum levels of Aβ_42_, measured in two APP21 homozygous rats, were 135 ± 64 pg/ml. Only one out of eleven APP31 rats had measurable levels of Aβ_42_.

**Table 2:** Serum Aβ_40 _levels of homozygous and hemizygous APP21 and APP31 lines determined by ELISA. h?: homozygosity status not determined; he:hemizygous; ho: homozygous; SE: Standard error; N: number of rats included in the statistical test; P: P value. All rats in the APP21 line had measurable levels of Aβ_40 _in serum. The Aβ_40 _levels were out of range in 10 of 20 rats in the APP31 line.

	Aβ_40 _(pg/ml)	SE	N	Age, d	P
APP21 h?	466.2^A^	46.2	9	61	<.0001
APP21 he	298.2^A^	34.7	16	151 ± 81	<.0001
APP21 ho	486.0^A^	98.0	2	70	<.0001
APP31 h?	93.2^B^	33.6	17	81 ± 42	0.0083
APP31 ho	0.0^B^	80.0	3	107	1

### Brain Immunohistochemistry

**Figure 6:**
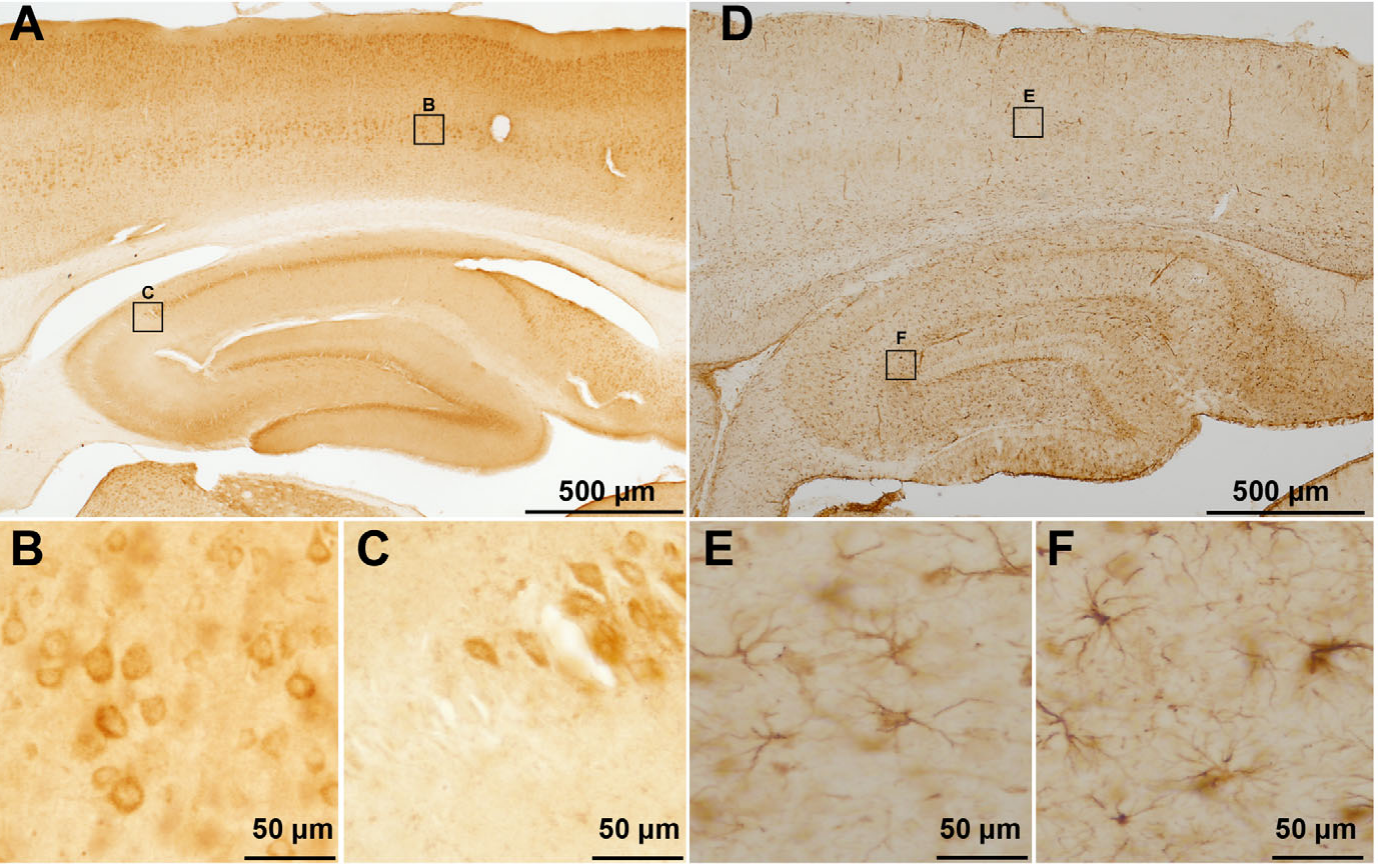
Human APP_Sw/Ind _expression in transgenic rat brain. **A**. A low power (2×) micrograph demonstrates widespread expression of human APP in the neocortex and hippocampus. Higher power (20×) images show punctate cytoplasmic staining in large cortical pyramidal neurons (**B**) and hippocampal pyramidal neurons (**C**). APP staining appears to demarcate the margins of CA2 pyramidal neurons, which were largely devoid of staining (**C**). Low power (2×) micrograph of an adjacent section stained with an anti-GFAP antibody. GFAP staining reveals the presence of glia and astrocytes within the neocortex and hippocampus (**D**). Higher power (20×) images from the cortex (**E**) and hippocampal dentate gyrus (**F**) are shown for comparison to (B-C) and illustrate that human APP_Sw/Ind _expression in occurring predominately in neurons.

Figure 6 shows a representative section from a two month-old homozygous male derived from founder #21. The low power micrograph (Fig. 6A) demonstrates widespread expression of human APP in the cortex and hippocampus detected by human APP-specific staining with antibody 6E10. The distribution of staining shows strong neuronal expression in neocortex, hippocampal dentate granule cells, and hippocampal pyramidal neurons. No staining was observed in control sections when primary antibody was omitted [see Additional file 1]. Higher power images show punctate cytoplasmic staining in large cortical pyramidal neurons (Fig. 6B) and hippocampal pyramidal cells (Fig. 6C). Unexpected selectivity was noted in the hippocampus, where intense staining was seen in CA3 and CA1 neurons, but 6E10 staining appeared to demarcate the margins of CA2, which was largely devoid of staining. High power images demonstrate the absence of human APP transgene expression in glial cells. Staining with an antibody directed against the glial fibrillary acidic protein (GFAP), a marker for astrocytes and the glia, demonstrates the absence of human APP transgene expression in glial cells (Fig. 6D) of the cortex (Fig. 6E) or hippocampus (Fig. 6F).

### APP Transgene Expression in C3-3 Mice

**Figure 7:**
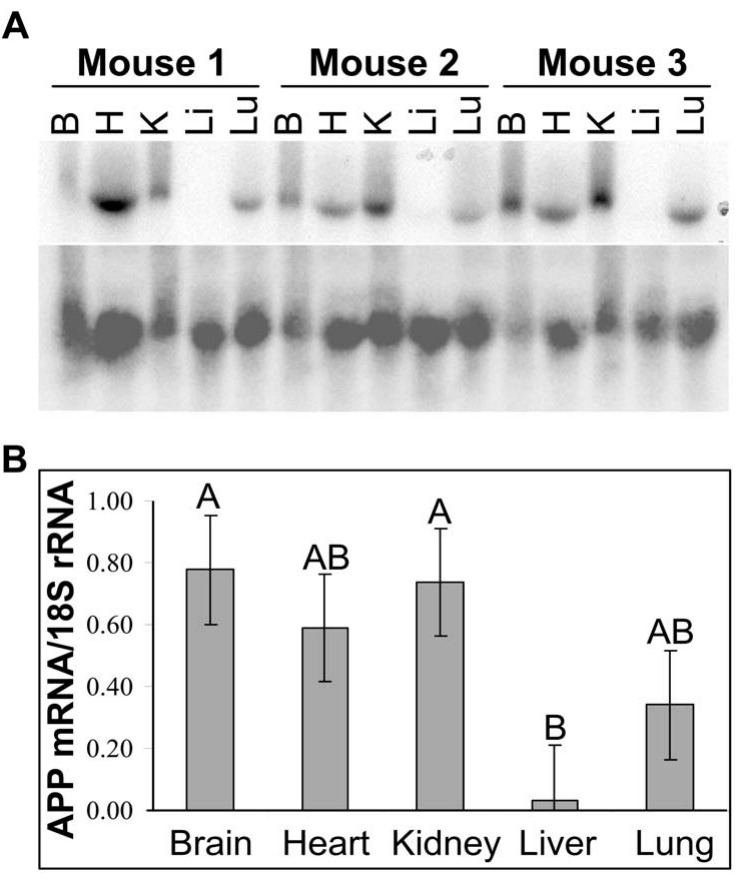
Gene expression analysis of APP-transgenic mice. **A**. Northern blot hybridization of total RNA from C3-3 APP-transgenic mouse tissues. Total RNA hybridized with a human APP probe (upper autoradiogram) prior to hybridization with 18S rRNA (lower autoradiogram). **B**. Gene expression differences among organs obtained from C3-3 mice. The expression of APP genes was normalized by dividing the net intensity of APP bands by the 18S rRNA bands. Different letters above the bars represent statistically significant (P < 0.05) expression levels. B: Brain, H: Heart, K: Kidney, Li: Liver, Lu: Lung.

Human APP cDNA expression in Tg C3-3 mice [17] was analyzed to determine if a different promoter, the prion protein promoter (PrP), would also drive the expression of the human APP cDNA in a tissue-specific manner similar to that observed in APP21 and APP31 Tg rats. Northern blotting results (Fig. 7) showed that transgenic APP expressed tissue-specifically in C3-3 mice as well. The transgenic APP expression was highest in brain, heart, kidney and lung, but very little APP expression was detected in the liver of C3-3 mice. Relative expression in brain, kidney, liver and lung was similar in APP21 and APP31 rats and C3-3 mice. However, in C3-3 mice, expression of APP in heart was similar to APP expression levels in brain, kidney and lung. Intriguingly, these two different Tg species (mouse and rat), in which the APP cDNA transgene is under the control of two unrelated promoters, show a similar pattern of tissue-specific expression

## Discussion

This study demonstrates the efficacy of generating inbred Tg rats using a lentiviral vector, which is a novel approach for generating Tg animals [18]. Despite dozens of Tg mouse models of AD-like pathology, few Tg rat lines are available for AD research [10-13]. Since the manifestation of specific genetic disorders in transgenic models is expected to be unique to each species and strain, it is essential to control phenotypic variations that stem from the genetic constitution of the background strain. Thus we chose the inbred Fischer 344 rat strain for transgenic production in order to minimize individual variation among transgenic rats. In transgenic mouse models of AD, Lehman et al. [8] reported that genetic background had a significant influence on the regulation of APP and Aβ deposition in Tg mice that were created on different genetic backgrounds. For our studies, we chose inbred Fischer 344 rats due to their well-defined genetics as well as their common usage in studies involving aging [19]. As Fischer 344 rats age, their brains are increasingly susceptible to oxidative stress, which is known to correlate with many neurodegenerative diseases, including AD. However, the approach that we employed to create germline Tg APP_Sw/Ind _lines using Fischer 344 rats can be equally applied to other strains, providing unrestricted opportunities to create disease models on various genetic backgrounds.

In this paper, we report the successful generation of APP_Sw/Ind _Tg Fischer 344 rats expressing human APP695 containing the Swedish and Indiana mutations under the control of the Ubi-C promoter using a lentiviral vector. The APP695 form was chosen because the predominant human APP isoform expressed in neurons of the central nervous system (CNS) is the APP695 isoform [20]. Additionally, we chose to construct the double-mutant APP695 transgene to facilitate comparisons with existing APP695 transgenic mouse and rat models, particularly the Tg2576 mice reported by Hsiao et al. [21].

Long-lasting neuronal expression of transgenes is an important consideration in modeling neurodegenerative diseases such as AD. Our earlier efforts to create Tg APP-and PS1-overexpressing SD rats under the control of the CMV promoter using lentiviral vectors were successful in terms of efficient gene delivery. However, the Tg rats did not have the desired level of gene product as assessed by ELISA and Western blot analysis, even at 10 months of age (unpublished data). This lack of expression may be attributable to the phenomenon of gene silencing, which has previously been observed with a number of promoters, including CMV. In the present study, the easily detectable eGFP reporter gene was used to compare eGFP expression after stereotaxic injection of lentiviral vectors containing various promoters. In these studies, CMV, PDGF, and Ubi-C promoters all drove high-level expression of eGFP *in-vitro*. However, when tested *in vivo *after stereotaxic injection or creation of Tg animals, CMV-eGFP and PDGF-eGFP expression decreased dramatically over time, whereas eGFP expression remained strong for up to 13 months when driven by the Ubi-C promoter. Characterization of transgene expression in rat brains revealed long-lasting and selective neuronal expression of genes driven by the Ubi-C promoter. Stereotaxic injections were used to qualitatively screen CMV, UbiC, and PDGF promoters for ability to drive eGFP transgene expression within the hippocampus. The choice of SD rats was based on the use of this strain in the stereotaxic mapping of the rat brain by Paxinos & Watson [22]. The dentate gyrus injection coordinates extrapolated from the atlas have been experimentally verified in our laboratory using SD rats and therefore we used SD rats to examine eGFP expression from the CMV, UbiC, and PDGF promoters. The goal of the promoter comparisons was not to characterize the expression patterns of the CMV, UbiC, and PDGF promoter in the CNS, which has previously been well documented, but rather to show qualitative examples of eGFP expression within the hippocampus following focal injection of lentivirus. Stereotaxic injections were performed in dentate gyrus to allow qualitative assessment of gene expression within the hippocampal formation, a region particularly vulnerable to neuropathological insult in AD. We determined that the eGFP signal consistently appears to be both nuclear and cytoplasmic *in vitro *and *in vivo*, regardless of the method of transgene delivery or type of promoter. Similar results were obtained by Wei et al. [23], who showed that eGFP diffused bidirectionally via the nuclear pore complex across the nuclear envelope.

To our knowledge, this is the first inbred, APP-transgenic rat model of AD that has substantial quantities of Aβ in serum. Prior to the generation of APP21 and APP31 transgenic rat strains, a Fischer 344 inbred AD model expressing APP (TgAPP_Sw_) was reported by Ruiz-Opazo et al. [13]. However, APP expression in these TgAPP_Sw _rats was only 56% greater than in WT rats. In addition, APP-transgenic, outbred Wistar rats expressed 2.5 times more APP in hippocampus than did control rats [24] In the current paper, we report 2.9 times greater APP expression in the brains of inbred Fischer 344 rats than in WT controls. Due to the higher APP expression, APP21 rats could be useful models for examining the underlying mechanisms of AD progression and for developing and testing potential therapies for AD.

Several mouse models have been generated to study the effects of APP mutations. Hsia et al. [25] generated Tg APP_Sw/Ind _mice. The characterization of these Tg mice indicated that the neurotoxic effects of Aβ may not require plaque formation. APP23 mice express 7-fold more APP_Sw _than endogenous mouse APP and develop Aβ deposits at 6 months of age [26]. Tg APP mice were generated using tissue-specific promoters such as enolase, platelet derived growth factor, and Thy-1 [27-29]. Similarly, we show that promoter choice significantly influences the expression of the transgene, such that Ubi-C is superior to CMV and PDGF in rats. The present study describes the generation of APP Tg Fischer 344 rats under the control of the Ubi-C promoter, which drives transcription in all tissues relatively stably. The expression of APP_Sw/Ind _transgenic mRNA was detectable in all tissues analyzed for both the APP21 and APP31 lines. The expression of transgene was greater in the APP21 line than in the APP31 line. Since these Tg rats were generated as models of AD-like pathology, the expression level of the transgene is particularly important. The APP21 line showed 3 times greater cerebral APP expression compared to WT rats. The expression of brain APP in the APP31 line was about half that of the APP21 line. In addition, serum Aβ_40 _levels corroborate these findings. The APP21 line had significantly greater human Aβ_40 _than did the APP31 line. In transgenic mouse models, the expression of APP has reached levels as high as 10-fold greater than endogenous APP [28]. However, protein expression level is not the only determinant of AD phenotype, as lower expression of mutant forms of APP can induce early and robust deposition of Aβ in brain parenchyma and/or vasculature [29,30].

While the anticipation of stable transgene expression was a key consideration in selecting the Ubi-C promoter for our studies, selective neuronal expression driven by this promoter was unexpected. Our observations in brain and primary cultures from eGFP-transgenic SD rats confirmed the highly preferential expression of eGFP in neurons versus glial cells (Fig. 2). Potentially even greater levels of selectivity are suggested by our characterization of APP_Sw/Ind _expression in transgenic Fischer 344 rats. In these animals, human APP was strongly expressed in neurons, but within the hippocampus, there was a strong demarcation based on the intensity of immunostaining in the pyramidal cell layer between CA1 and CA2 (Fig. 6). The basis for this selectivity is unclear, and additional studies will be required to fully evaluate the distribution of Ubi-C promoter-driven transgene expression in brain.

We chose Northern blot analysis to assess gene expression in APP21 and APP31 transgenic and WT rats and C3-3 mice. This allowed us to confirm the size of the complete transcript in the transgenic animals. Samples collected from brain yield a higher band for APP mRNA and 18S rRNA for both rat and mouse samples. Since both APP mRNA and 18S rRNA migration was retarded in brain RNA samples compared to the rest of the organs, we suspect that brain RNA samples contain residual substances that impede the migration of brain RNA. These could be residual lipids, as the brain contains more fat compared to the other organs analyzed.

Since the sequences of human and rat APP are highly similar, the APP probe used for Northern blot analysis did not distinguish between human and rat APP. This enabled comparison of native rat APP- and human APP-transgene expression. The transgene as well as the endogenous rat APP gene expression patterns showed significant tissue-specificity. Interestingly, both the human APP transgene and endogenous rat APP mRNA were more abundant in kidney and lung than in heart and liver. Although tissue-specific expression of APP in humans was reported previously [31], tissue-specific expression of the APP transgene was unexpected in the transgenic rats because the Ubi-C promoter drives expression of the human APP transgene in this construct. We have generated purinergic receptor Y2- (P2RY2) transgenic rats using the same vector backbone, and expression of the P2RY2 transgene driven by the Ubi-C promoter did not show tissue-specificity (unpublished data).

Tissue specific expression of APP transgene can be due to APP mRNA stability, RNA silencing or transcription regulatory elements within APP cDNA. The 3'UTRs of human, rat and transgenic APP are substantially different which reduces the possibility of tissue specific differences in APP mRNA stability. In addition, we were unable to find a candidate sequence for RNA silencing within the rat or human genome. Thus, regulation through enhancer or silencer elements within the APP cDNA is a possible explanation for tissue-specific expression regardless of the promoter that drives the expression of APP. A recent publication by Collin and Martens [32] supports the presence of transcription regulatory role of APP cDNA.

In order to confirm tissue-specific expression of APP, we analyzed APP transgene expression in C3-3 transgenic mice, which is driven by the ubiquitously expressed prion protein promoter [17]. Expression levels of endogenous prion protein are similar in various tissues. We suggest that the similarity of tissue-specific expression patterns of APP transgenes that are driven by two different promoters (Ubi-C and PrP) in two different species (rat vs. mouse) strongly supports the presence of transcription regulatory elements within the APP cDNA. The temporal and spatial expression differences in APP-transgenic mice have been attributed to the promoters (PDGF, and Thy-1) used to drive expression [33]. However, we believe that the changes in APP expression patterns shown in our study cannot be explained by the promoter because of the stable expression driven by the Ubi-C promoter in most tissues. We hypothesize that elements within the cDNA sequence may regulate the expression of APP tissue-specifically. Identification of these elements might ultimately broaden treatment options for AD. In conclusion, these APP-transgenic rats could be a useful model in which to study the regulation of APP expression as well as pathogenic mechanisms in AD.

## Conclusion

This study shows the high efficiency of establishing stable, inbred germline transgenic rats by lentiviral gene delivery. The APP21 rats, which express high levels of human APP, could be a valuable model of AD. Furthermore, the tissue-specific expression of the APP transgene indicates the presence of regulatory elements within APP cDNA that could be a useful target for AD treatment or prevention. Our ongoing studies are aimed at intensive pathological and behavioral characterization of these unique APP-transgenic rats.

## Methods

### Construct Design and Preparation of VSV-G Pseudotyped Lentivirus

**Figure 8:**
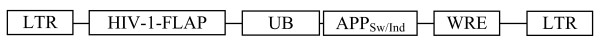
DNA construct of pLVU-APP_Sw/Ind_. LTR: long terminal repeat; UB: ubiquitin-C promoter; APP: human amyloid precursor protein; WRE: woodchuck hepatitis virus post-transcriptional regulatory element.

Human APP (695 amino acid isoform) was mutagenized using the QuickChange II XL site-directed mutagenesis kit (Stratagene, La Jolla, CA) to incorporate the Swedish double missense mutation (K595M/N596L) and Indiana single missense mutation (V642F). The APP sequence was cloned into the pLVU-eGFP cassette [18] in place of the eGFP coding sequence. The new vector was designated as pLVU-APP_Sw/Ind_. The APP transcription was under the control of the Ubi-C promoter. In brief, pLVU-APP_Sw/Ind _is a self-inactivating vector, composed of the woodchuck hepatitis virus post-transcriptional regulatory element (WRE) to increase transcription level and minimize position effect. Additionally, an HIV-1-flap element was inserted between the 5'LTR and the internal promoter, which increases titer (Fig. 8). Preparation of VSV-G Pseudotyped Lentivirus was as described [18].

### Promoter Characterization

To select an advantageous promoter to drive transgene expression in brain, lentiviruses were constructed using Ubi-C, CMV, and PDGF promoters to drive expression of eGFP. Lentiviruses were stereotaxically injected into the hippocampus of SD rats. Qualitative comparisons of eGFP expression were made 3 weeks and 3 months following injection. To characterize Ubi-C promoter driven transgene expression, eGFP-transgenic SD rats were created as described below and analyzed by confocal microscopy.

### Cell Culture

Rat primary E18 cortical cultures were established from timed-pregnant female SD females that were bred to eGFP Tg males. Mixed neuronal and glial cultures were plated on polylysine-coated coverslips and processed for immunofluorescence. Neurons were identified by immunostaining with anti-beta III tubulin (Promega, Madison, WI), and astrocytes were labeled with anti-GFAP (Dako, Carpinteria, CA).

### Stereotaxic Injection

Lentiviruses were injected stereotaxically into the hippocampus of adult male SD rats weighing 250–300 g. Rats were anesthetized by IM injection of ketamine-xylazine and positioned in a Kopf Small Animal Stereotaxic Instrument (David Kopf Instruments, Tujunga, California). One microliter of lentivirus was injected bilaterally into the dentate gyrus at the following coordinates with respect to bregma: AP -3.30 mm, ML +/- 1.6 mm, DV 3.2 mm. A 5-μl Hamilton syringe was used to deliver virus at a rate of 150 nl/min.

### Zygote Collection, Microinjection of Lentiviral Vector and Embryo Transfer

Fischer 344 female rats (28–30 day-old) were purchased from Harlan Sprague Dawley Inc. (Indianapolis, IN) and superovulated using follicle-stimulating hormone and luteinizing hormone; zygotes were collected after mating [34]. Morphologically normal zygotes having two pronuclei were used for lentiviral vector injections as described earlier [18]. Lentiviral vector-injected zygotes were then transferred into the oviducts of 8–10 week-old pseudopregnant recipient rats.

### Breeding of Transgenic Rats

Fischer 344 Tg founders were mated with WT Fischer 344 rats to determine germline transgenesis. After germline transgenesis was confirmed, rats from the F1 generation were used to generate homozygous Tg rats. Homozygosity was initially determined by Southern blot hybridization. Conventional matings were used to confirm the homozygosity of Tg rats. All animal studies were performed in accordance with the University of Missouri's Animal Care and Use Committee guidelines and the ILAR Guide for the Care and Use of Laboratory Animals. The rats were housed in conventional cages at 20–25°C in a controlled lighting environment and provided free access to water and standard pelleted rodent chow. Rats were euthanized with an inhaled overdose of CO_2_.

### Genomic DNA Isolation and Polymerase Chain Reaction

Genomic DNA from tail-snips was isolated using the Wizard Genomic DNA Purification Kit (Promega, Madison, WI). PCR was used for screening of Tg rats. Primers annealing the Ubi-C promoter (TGTCCGCTAAATTCTGGCCGTT) and APP transgene (ATTTCGAGCATGTGCGCATGGT) were used in the PCR reactions. The 50 μl reactions were carried out using 50 ng genomic DNA, 100 ng of each primer and 1.5 U Biolase taq (Bioline, Randolph, MA). The amplified PCR products were size-separated through 1% agarose gel and stained with ethidium bromide for visualization.

### Southern blot Analysis

Southern blot analysis was done to determine the copy number as well as homozygosity of the Tg rats. Genomic DNA was digested with BamHI or EcoRI, which only cut the junction of the human Ubi-C promoter and APP transgenes or the junction of APP and WRE transgenes, respectively. The digestion products were size-separated through a 0.8% agarose gel and transferred to a Genescreen plus membrane (Perkin Elmer, Wellesley, MA) overnight. The 773 bp APP probe template was prepared by amplification of pLVU-APP using forward (TGTTGCCCACTGGCTGAAGAAA) and reverse (ATTTCGAGCATGTGCGCATGGT) primers. The ^32^P labeled probe was generated using the probe template, Ready-To-Go DNA Labeling Beads (Amersham Biosciences, Piscataway, NJ) and [α-^32^P]-dCTP (Perkin Elmer, Wellesley, MA). The membranes were prehybridized in 10% dextran sulfate, 6× SSC, 1% SDS for 2 hours and hybridized using the ^32^P labeled probe overnight before exposing to BioMax MS-1 Autoradiography Film (Kodak, Rochester, NY). To determine homozygosity, the membranes were hybridized with P2RY2 probe following hybridization with the APP probe. P2RY2 was used as a positive control for homozygosity to correct for pipetting differences between the samples. The intensity of bands was determined using Kodak 1D v 3.6.3 software (New Haven, CT).

### Northern blot Analysis

Total RNA was isolated using Trisure (Bioline, CA) from APP21 (n = 2), APP31 (n = 3), and WT (n = 3) rats as well as APP-transgenic C3-3 mice (n = 3). Brain, heart, kidney, liver and lung tissues were used in Northern blot analysis. Total RNA was size-separated through 1% agarose gel before transferring to the Genescreen plus membrane overnight. The membrane was prehybridized in 10% dextran sulfate, 5× SSPE, 50% formamide, 5× Denhardt's, 1% SDS at 42°C for 6 hours prior to hybridization with the APP probe overnight. Membranes were subsequently hybridized with an 18S rRNA probe to correct for pipetting differences. Intensity of bands was determined using Kodak 1D v 3.6.3 software (New Haven, CT).

### ELISA Measurement of Aβ_40 _and Aβ_42_

To screen for transgene expression, serum samples were collected and assayed for Aβ_40 _and Aβ_42 _using commercial ELISA kits (Genetics Company, Schlieren, Switzerland). Serum samples were diluted in assay buffer and processed according to the manufacturer's recommended protocols. Briefly, samples and standards were incubated in capture wells overnight at 4°C with biotinylated Aβ_40 _or Aβ_42_-specific antibody. After several rinses, the enzyme-conjugated detection reagent was added to the wells for 30 minutes. After additional rinses, wells were incubated with the chromogen solution for 30 minutes at room temperature, shielded from light. After addition of the stop solution, the wells were read for absorption at 450 nm, and Aβ concentration in the samples was calculated from standard curves.

### Brain Immunohistochemistry and Confocal Microscopy

Hemi-brains of human APP Tg rats were immersion-fixed with 4% paraformaldehyde for 3 hours at 4°C, then cryoprotected in 30% sucrose prior to sectioning on a freezing-sliding microtome to obtain 50 μm-thick sagittal sections. Immunohistochemical processing was performed using free-floating sections and immunoperoxidase methods. Sections were treated with hydrogen peroxide, washed in Tris buffer, blocked with normal serum, and incubated with human-specific 6E10 mouse monoclonal anti-APP antibody (Signet; Dedham, MA) or rabbit polyclonal anti-GFAP (DAKO; Carpinteria, CA). On day 2, sections were incubated with biotinylated secondary antibody followed by the avidin-biotin-peroxidase complex for 1 hour at 4°C (Vector Elite ABC kit; Vector Laboratories, Burlingame, CA). Immunoreactivity was visualized with 3, 3'-diaminobenzidine tetrahydrochloride. For high-resolution light microscopic localization, confocal images were captured on a Zeiss LSM510-NLO microscope using 1 μm optical sections. For promoter comparisons, sections were immunostained with rabbit polyclonal anti-GFAP (DAKO; Carpinteria, CA) to label glia and mouse monoclonal anti-beta III tubulin antibody to (Promega; Madison, WI) to identify neurons then eGFP was directly visualized using a Spot Flex digital cameral (Diagnostic Instruments, Sterling Heights, MI) attached to a Leica DMLB microscope (Wetzlar, Germany). For qualitative comparison of eGFP intensity, equivalent fields from injection sites were captured using identical objectives and microscope/camera settings.

### Statistical Analysis

Statistical analysis was performed using general linear models of SAS version 9.1 (Cary, NC) to determine the gene expression differences in APP21, APP31 and WT rats, as well as differences in serum Aβ concentrations.

## Competing interests

The author(s) declare that they have no competing interests.

## Authors' contributions

CA was responsible for colony management, genotypic and phenotypic characterization of transgenic rats, statistical analysis of data and writing the paper, JJL and JJF generated lentiviral vectors, performed stereotaxic injections, ELISA and brain immunohistochemisty, YA generated transgenic rats, and CA, LCW, AIL, AWSC, JJL, YA designed the research and contributed to the analysis and presentation of the data. All authors read and approved the final manuscript.

## Supplementary Material

Additional file 1 Control immunohistochemistry without primary antibody for 6E10 staining. Low power (2×) micrograph demonstrates the lack of non-specific staining in neocortex and hippocampus with biotynalated goat anti-mouse secondary antibody (Vector Laboratories: Burlingame, CA) following no primary negative control for immunohistochemistry with human-specific APP mouse monoclonal antibody 6E10 (Signet; Dedham, MA). Click here for file
