# Supplementary figures and images for: Development of transgenic rats producing human β-amyloid precursor protein as a model for Alzheimer's disease: Transgene and endogenous APP genes are regulated tissue-specifically
Source: BMC Neurosci. 2008 Feb 26;9:28. doi: 10.1186/1471-2202-9-28 (PMC2268936; doi:10.1186/1471-2202-9-28)

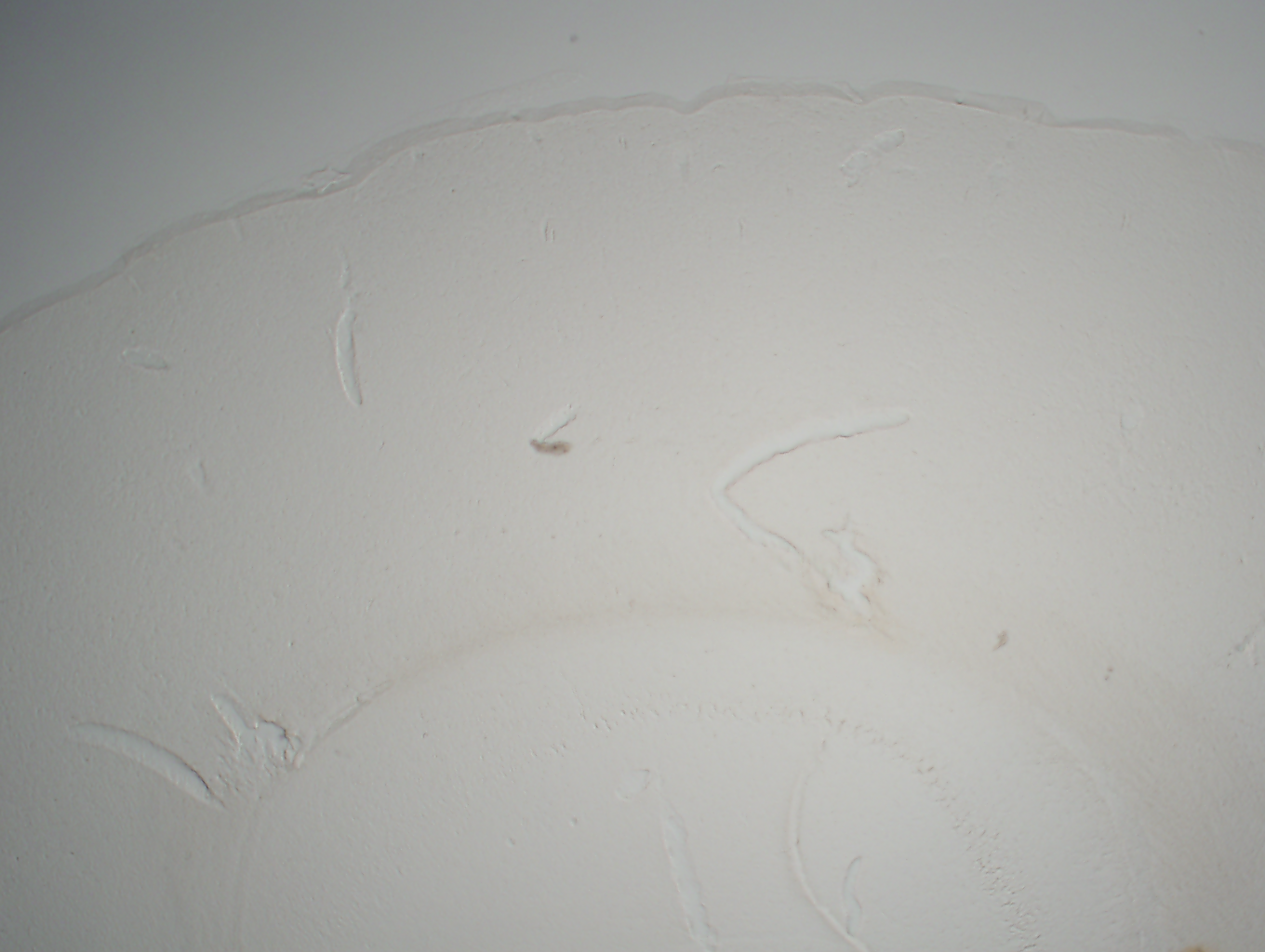

Supplement: Additional file 1 — Control immunohistochemistry without primary antibody for 6E10 staining. Low power (2×) micrograph demonstrates the lack of non-specific staining in neocortex and hippocampus with biotynalated goat anti-mouse secondary antibody (Vector Laboratories: Burlingame, CA) following no primary negative control for immunohistochemistry with human-specific APP mouse monoclonal antibody 6E10 (Signet; Dedham, MA). [file 1471-2202-9-28-S1.doc]
